# Elevated Salivary Inflammatory Biomarkers are Associated with SARS-CoV-2 Infection Severity

**DOI:** 10.1155/2022/1543918

**Published:** 2022-08-04

**Authors:** Hanadi Abdullah Alwafi, Soad Shaker Ali, Sunil Babu Kotha, Layla Waleed Abuljadayel, Maha Ibrahim, Ibrahim Rashad Noor Elahi, Hebah Abdullah Alwafi, Mohammed S. Almuhayawi, Matthew D. Finkelman, Nagla A. El-Shitany

**Affiliations:** ^1^Department of Preventive Dentistry, College of Dentistry, Riyadh Elm University, Riyadh, Saudi Arabia; ^2^Department of Histology and Cell Biology, Faculty of Medicine, Assiut University, Assiut, Egypt; ^3^Yousef Abdullatif Jameel Chair of Prophetic Medicine Application, Faculty of Medicine, King Abdulaziz University, Jeddah, Saudi Arabia; ^4^Department of Pediatric and Preventive Dentistry, Sharad Pawar Dental College and Hospital, Datta Meghe Institute of Medical Sciences, Sawangi (Meghe), Maharashtra, India; ^5^Department of Dental Public Health, Faculty of Dentistry, King Abdulaziz University, Jeddah, Saudi Arabia; ^6^Department of Hematology, MSF for Medical Research and Development, Jeddah, Saudi Arabia; ^7^Department of Public Health, Directorate of Health Affairs, Jeddah, Saudi Arabia; ^8^Security Force Hospital, Dammam, Saudi Arabia; ^9^Department of Medical Microbiology and Parasitology, Faculty of Medicine, King Abdulaziz University, Jeddah, Saudi Arabia; ^10^Division of Biostatistics and Experimental Design, Department of Public Health and Community Service, Tufts University School of Dental Medicine, Boston, MA, USA; ^11^Department of Pharmacology and Toxicology, Faculty of Pharmacy, Tanta University, Tanta, Egypt

## Abstract

High levels of inflammatory cytokines in serum have been reported in patients with severe SARS-CoV-2 infection. There is growing interest in recognizing the role of inflammatory biomarkers in saliva in diagnosing systemic diseases. This study assumed that estimating biomarkers in saliva samples from patients infected with SARS-CoV-2 would distinguish between mild and severe cases. Saliva was collected from 142 controls and 158 SARS-CoV-2 patients (mild 72 and severe 86) to measure interleukin-6 (IL-6), C-reactive protein (CRP), and C-X-C motif chemokine ligand-10 (CXCL-10). IL-6 and CXCL-10 were significantly increased in patients with mild and severe SARS-CoV-2 infections. CRP was significantly increased only in severe SARS-CoV-2 cases. All biomarkers were significantly higher in severe cases than in mild cases (*p* < 0.001). Among patients with SARS-CoV-2 infection, men showed significantly higher CRP and CXCL-10 levels than females (*p* < 0.01 and 0.05, respectively). In addition, elderly patients (40–80 years) had significantly higher IL-6, CRP, and CXCL-10 (*p* < 0.001). Patients with diabetes and hypertension showed elevated IL-6, CRP, and CXCL-10 (*p* < 0.001). There was a significant positive correlation between IL-6, CRP, CXCL-10, and between age, IL-6, CRP, and CXCL-10. Saliva may have a future value in measuring the inflammatory biomarkers associated with the severity of SARS-CoV2 infection and therapeutic monitoring.

## 1. Introduction

Coronavirus disease 2019 (COVID-19) is a fatal disease caused by the unique SARS-CoV-2 virus spreading throughout the world [1]. The disease manifests itself in a variety of symptoms and degrees of severity. While most SARS-CoV-2 virus infections are asymptomatic and mild, the full spectrum of illnesses encompasses more severe illnesses, ranging from moderate to severe, necessitating increasing medical intervention, and is potentially fatal [2–5]. In severe cases, fast clinical worsening is common, and the serious stage of the disease is characterized primarily by a systemic hyperinflammation known as a “cytokine storm” [6, 7]. Inflammatory cytokines and chemokines (interleukin (IL)-6, C-X-C motif chemokine ligand 10 (CXCL-10), and IL-10), inflammasome (IL-18 andIL-1), and interferons (interferon (IFN)-*α*, IFN-*γ*, and IFN-*λ*) are overexpressed in patients with severe SARS-CoV-2 infection [8–10]. Recent research has shown that C-reactive protein (CRP) concentration is higher in SARS-CoV-2-infected patients and may be related to the severity and progression of the disease [11, 12]. The inflammatory biomarkers, including IL-6, CRP, and CXCL-10, could help clinicians better understand disease progression and therapeutic responses to specific therapies. Until now, these inflammatory biomarkers were only examined in serum samples of SARS-CoV-2-infected patients [13].

There is increasing interest in recognizing the role of inflammatory biomarkers in saliva in diagnosing systemic diseases [14]. Saliva provides several advantages over blood as a noninvasive alternative biological fluid, including ease of collection from complex patients, repeatability, cost-effectiveness, the lack of a need for rapid sample processing, and the flexibility to collect samples anywhere [15]. Furthermore, collecting saliva on one's own minimizes the risk of SARS-CoV-2 infection spreading to healthcare workers.

According to Adeoye and Thomson's theory, biomarkers for SARS-CoV-2 infection outcome detection could be secreted in saliva via systemic- and local-based outflow mechanisms [16]. This study hypothesized that estimating biomarkers in saliva samples from SARS-CoV-2-infected patients would distinguish between mild and severe cases. Therefore, the study aimed to identify and compare salivary IL-6, CRP, and CXCL-10 concentrations in mild and severe SARS-CoV-2-infected patients and compare them with the results of a group of healthy participants.

## 2. Materials and Methods

### 2.1. Participants

This study was approved by the Saudi Ministry of Health (MOH) (Institutional Review Board of Research and Studies Department, Jeddah Health Affairs, Saudi Arabia). The IRB approval number is A00981. This study included 142 healthy participants who constituted the noninfected participants and 158 SARS-CoV-2-infected patients. The SARS-CoV-2-infected patients were further divided into the mildly infected group (86 patients) and the severe infected group (72 patients). Patients were classified according to the MOH classification during the SARS-CoV-2 virus pandemic guidelines based on whether they only needed supportive measures (mild) or monitoring and/or intervention in the intensive care unit (ICU) (severe). Using the mean and standard deviation of IL-6 in healthy and SARS-CoV-2-infected patients and a power of 80% at a 5% level of significance, the number of individuals was calculated. The data analysis was carried out using ClinCalc statistic analyzer software (https://clincalc.com/stats/samplesize.aspx) [17].

Mild SARS-CoV-2-infected patients were recruited from COVID-19 isolation centers in Jeddah, Saudi Arabia. On the other hand, severe SARS-CoV-2-infected patients were recruited from many local government medical hospitals in Jeddah, Saudi Arabia (The Field Hospital, King Fahad General Hospital, King Abdullah Medical Complex Hospital, and East Jeddah General Hospital). Before the study began, all participants signed an informed consent form. The research was carried out between September 1, 2020, and September 1, 2021.

### 2.2. Inclusion Criteria

All included participants were unvaccinated. Non-SARS-CoV-2-infected participants were not previously infected with the coronavirus. Negative PCR results confirmed non-SARS-CoV-2-infected participants. Positive PCR results confirmed patients infected with SARS-CoV-2. The PCR was done using either nasopharyngeal or oropharyngeal swabs.

### 2.3. Exclusion Criteria

Participants were vaccinated against the SARS-CoV-2 virus, under 18 years of age, or had autoimmune disease, periodontitis, oral mucosal diseases, and oral cancer. Patients treated with any drug that affects IL-6, CXCL-10, and CRP.

### 2.4. Collection of Demographic and Clinical Data

Demographic and baseline clinical data of all participants, including nationality, age, gender, weight, height, and history of any chronic disease, were collected through a form created using the SurveyMonkey website and delivered through each patient's WhatsApp number.

### 2.5. Collection of Saliva Samples

All participants were asked to abstain from smoking, eating, drinking, and oral hygiene for at least one hour before sample collection. According to preestablished protocols of [15, 18], saliva samples were taken between 7 and 9 a.m. using the passive drooling procedure. Approximately 2 ml of total unstimulated saliva was collected. The samples were immediately frozen at −20°C and then stored at −80°C. To remove insoluble material and cell debris, the saliva samples were centrifuged at 3000 rpm and 4°C for 10 min. All experiments were carried out on the supernatants.

### 2.6. Measurement of Salivary Concentrations of IL-6, CRP, and CXCL-10

IL-6, CRP, and CXCL-10 concentrations in saliva were measured with the fully automatic ELISA DSX best 2000® microtiter plate. ELISA kits for IL-6 Cat. No. MBS021993, for CRP Cat. No. MBS2505217, and for CXCL10 Cat. No. MBS175853, respectively. The test was repeated twice to ensure accuracy. All tests were performed according to the manufacturer's recommendations.

### 2.7. Statistical Analysis of Data

Concerning demographic and baseline clinical data, descriptive statistics were performed, and data were expressed as frequency (number and percentage). Chi-squared and Fisher's tests were used to compare the demographic and clinical data of mild and severe SARS-CoV-2-infected groups.

Concerning the IL-6, CRP, and CXCL-10 measurements, the normality of the distributions was evaluated using the Kolmogorov-Smirnov normality test. The ANOVA test was used to compare the results of the non-SARS-CoV-2-infected participants, mild and severe SARS-CoV-2-infected patients, followed by Tukey's multiple comparisons test. The unpaired *t*-test was used to compare the data of non-SARS-CoV-2-infected participants and all SARS-CoV-2-infected patients. Regression and Pearson correlation were also performed.

Statistical data evaluation was performed using Prism® (version 8.4.0, GraphPad Software Inc., La Jolla, CA, USA). The level of significance was established at *p* < 0.05.

## 3. Results

### 3.1. Demographic and Clinical Data of the Study Participants

The sample consisted of 3 different groups: non-SARS-CoV-2 (*n* = 142), mild SARS-CoV-2 (*n* = 72), and severe SARS-CoV-2 (*n* = 86). In each group, the majority of participants were Saudi Arabian citizens: non-SARS-CoV-2 (79.6%), mild SARS-CoV-2 (59.7%), and severe SARS-CoV-2 (51.2%). Females made up most of the non-SARS-CoV-2 group (53.5%). Men predominated in the mild and severe SARS-CoV-2-infected groups (58.3% and 87.2%, respectively). The majority of participants in the non-SARS-CoV-2 group and mild SARS-CoV-2 group were between the ages of 20 and 40 years old (50.7% and 69.5%, respectively). However, in the severe SARS-CoV-2 group, the age group of 40–60 years predominated (58%). In the non-SARS-CoV-2 group, 30.3% of participants were in the normal weight category, 42.3% were overweight, and 26.7% were in the obese category. In the mild SARS-CoV-2 group, these percentages were 34.8%, 30.7%, and 31.9%, respectively; in the severe COVID-19 group, they were 11.6%, 38.4%, and 45.3%, respectively. In the non-SARS-CoV-2 group, 76% had no chronic diseases, while 10.6% had diabetes, 9.6% hypertension, 3.5% asthma, and 6.3% other diseases. In the mild SARS-CoV-2 group, 66.7% had no chronic conditions, 8.3% had diabetes, 9.7% had hypertension, 12.5% had asthma, and 16.7% had other ailments. In the severe SARS-CoV-2 group, 25.6% had no chronic diseases, 51.2% had diabetes mellitus (DM), 37.2% had hypertension (HT), 14% had asthma, and 20.9% had other conditions.  Table 1 shows a significant increase in males (*p* < 0.001), age between 40 and 80 years (*p* < 0.001), overweight and obese (*p* < 0.01), diabetics (*p* < 0.001), and hypertensive (*p* < 0.001) patients in severe SARS-CoV-2 group compared to mild SARS-CoV-2 group (Table 1).

### 3.2. Concentrations of IL-6, CRP, and CXCL-10 in Saliva and Their Correlations

**Figure 1:**
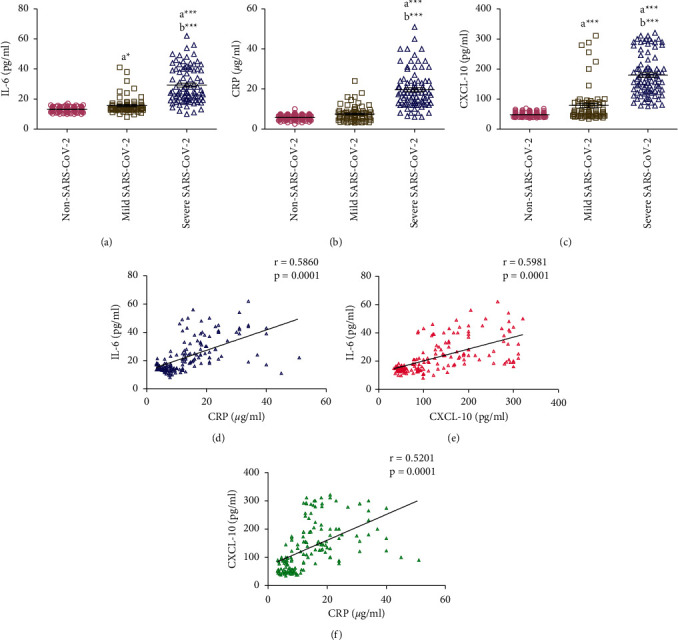
(a-c) Comparison of salivary concentrations of IL-6, CRP, and CXCL-10 between non-SARS-CoV-2 (*n* = 142), mild SARS-CoV-2 (*n* = 72), and severe SARS-CoV-2 (*n* = 86) participants. Data were graphed as a scatter plot vertical with mean ± SE. ^a^Significant difference compared to non-SARS-CoV-2 participants. ^b^Significant difference compared to mild SARS-CoV-2 patients. ^*∗*^*p* < 0.05, ^*∗∗∗*^*p* < 0.001. (d-f) Correlation between salivary IL-6, CRP, and CXCL-10 measured in SARS-CoV-2 patients (*n* = 158).

Mild SARS-CoV-2 patients showed significantly increased saliva IL-6 (*p* < 0.05) and CXCL-10 (*p* < 0.001) concentrations compared to non-SARS-CoV-2 participants. No significant difference was noted in saliva CRP concentration between mild SARS-CoV-2 patients and non-SARS-CoV-2 participants. Patients with severe SARS-CoV-2 had significantly higher (*p* < 0.001) saliva concentrations of IL-6, CRP, and CXCL-10 compared with non-SARS-CoV-2 and mild SARS-CoV-2 participants (Figures 1(a)–1(c)). Pearson's correlation was performed to assess the correlation between the measured inflammatory biomarkers using the data collected from all SARS-CoV-2 patients (mild and severe). A significant moderate positive correlation was found between IL-6 and CRP (*r* = 0.59, *p* < 0.001), IL-6 and CXCL-10 (*r* = 0.60, *p* < 0.001), and CXCL-10 and CRP (*r* = 0.5201, *p* < 0.001) (Figures 1(d))–1(f)).

### 3.3. Effect of Gender on Concentrations of Salivary IL-6, CRP, and CXCL-10

**Figure 2:**
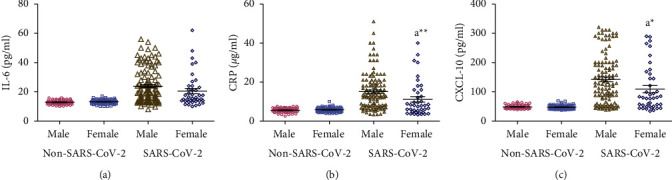
(a-c) Effect of gender on concentrations of salivary IL-6, CRP, and CXCL-10 measured in non-SARS-CoV-2 (*n* = 142) and SARS-CoV-2 (*n* = 158) participants. Data were graphed as a scatter plot vertical with mean ± SE. ^a^Significant difference compared to male SARS-CoV-2 patients. ^*∗*^*p* < 0.05 and ^*∗∗*^*p* < 0.01.

There were no significant differences in the salivary concentrations of IL-6, CRP, and CXCL-10 between male and female participants in the non-SARS-CoV-2 participants (Figures 2(a)–2(c)). Similarly, there was no significant difference in the salivary concentration of IL-6 between male and female SARS-CoV-2 patients (Figure 2(a)). However, compared with male SARS-CoV-2 patients, female SARS-CoV-2 patients had significantly lower salivary concentrations of CRP and CXCL-10 (*p* < 0.05) (Figures 2(b) and 2(c)).

Statistical analysis of data showed no significant differences between gender and salivary concentrations of IL-6, CRP, and CXCL-10 within the non-SARS-CoV-2, mild SARS-CoV-2, and severe SARS-CoV-2 groups (data not shown).

### 3.4. Effect of Age on Concentrations of Salivary IL-6, CRP, and CXCL-10 and Their Correlations

**Figure 3:**
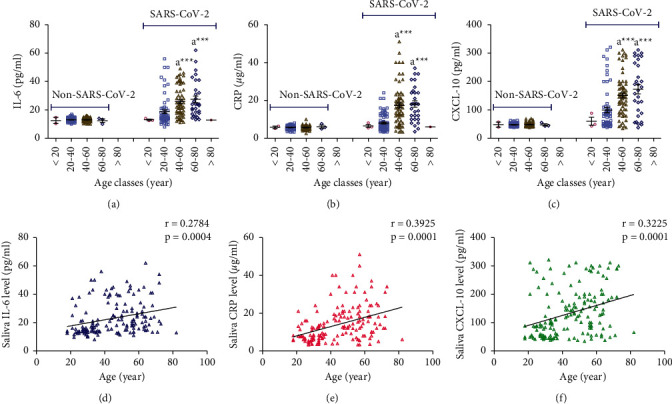
(a-c) Effect of age on concentrations of salivary IL-6, CRP, and CXCL-10 measured in non-SARS-CoV-2 (*n* = 142) and SARS-CoV-2 (*n* = 158) participants. Data were graphed as a scatter plot vertical with mean ± SE. (d-f) Correlation between age and salivary concentrations of IL-6, CRP, and CXCL-10 measured in SARS-CoV-2 patients (*n* = 158).

There were no significant differences in the salivary concentrations of IL-6, CRP, and CXCL-10 between the different age classes among the non-SARS-CoV-2 participants (Figure 3(a)–3(c)). On the other hand, salivary concentrations of IL-6, CRP, and CXCL-10 were significantly increased (*p* < 0.001) in SARS-CoV-2 patients aged 40–60 and 60–80 years compared to SARS-CoV-2 patients aged 20–40 years (Figures 3(a)–3(c)). Significant weak positive correlations were found between age and IL-6 (*r* = 0.28, *p* < 0.001), CRP (*r* = 0.39, *p* < 0.001), and CXCL-10 (*r* = 0.32, *p* < 0.001) calculated from data collected from all SARS-CoV-2 patients (mild and severe) (Figures 3(d)–3(f)).

Statistical analysis of data showed no significant differences between age and salivary concentrations of IL-6, CRP, and CXCL-10 within the non-SARS-CoV-2, mild SARS-CoV-2, and severe SARS-CoV-2 groups (data not shown).

### 3.5. Effect of BMI on Concentrations of Salivary IL-6, CRP, and CXCL-10 and Their Correlations

**Figure 4:**
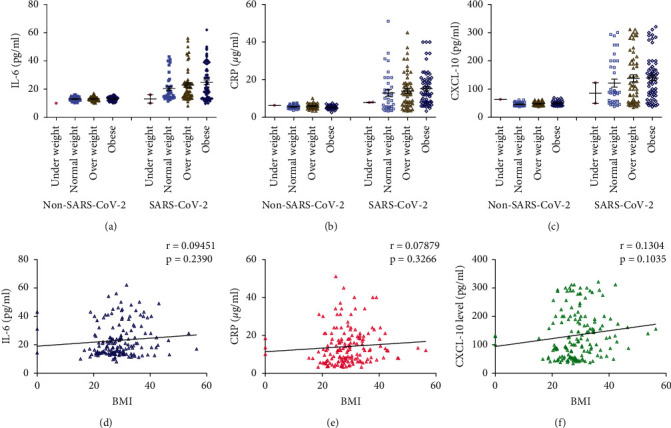
(a-c) Effect of body mass index (BMI) on concentrations of salivary IL-6, CRP, and CXCL-10 measured in non-SARS-CoV-2 (*n* = 142) and SARS-CoV-2 (*n* = 158) participants. Data were graphed as a scatter plot vertical with mean ± SE. (d-f) Correlation between BMI and salivary concentrations of IL-6, CRP, and CXCL-10 measured in SARS-CoV-2 patients (*n* = 158).

There were no significant differences in the salivary concentrations of IL-6, CRP, and CXCL-10 between the normal weight, overweight, and obese participants within the non-SARS-CoV-2 and the SARS-CoV-2 participants (Figures 4(a)–4(c)). No significant correlation was found between BMI and IL-6 (*r* = 0.095, *p*=0.24), CRP (*r* = 0.079, *p*=0.33), and CXCL-10 (*r* = 0.13, *p*=0.10) calculated from data collected from all SARS-CoV-2 patients (mild and severe) (Figures 4(d)–4(f)).

Statistical analysis of data showed no significant differences between BMI and salivary concentrations of IL-6, CRP, and CXCL-10 within the non-SARS-CoV-2, mild SARS-CoV-2, and severe SARS-CoV-2 groups (data not shown).

### 3.6. Effect of DM on Concentrations of Salivary IL-6, CRP, and CXCL-10

**Figure 5:**
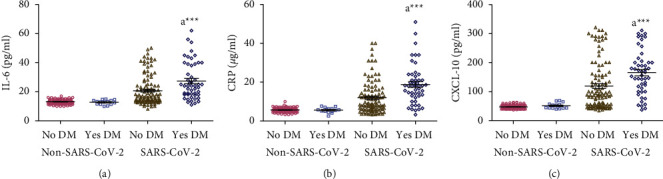
(a-c) Effect of diabetes mellitus (DM) on concentrations of salivary IL-6, CRP, and CXCL-10 measured in non-SARS-CoV-2 (*n* = 142) and SARS-CoV-2 (*n* = 158) participants. Data were graphed as a scatter plot vertical with mean ± SE. ^a^Significant difference compared to patients without DM. ^*∗∗∗*^*p* < 0.001.

There were no significant differences in the salivary concentrations of IL-6, CRP, and CXCL-10 between diabetic and nondiabetic participants in the non-SARS-CoV-2 participants. On the other hand, salivary concentrations of IL-6, CRP, and CXCL-10 were significantly increased (*p* < 0.001) in SARS-CoV-2 patients with DM compared to SARS-CoV-2 patients without DM (Figures 5(a)–5(c)).

Statistical analysis of data showed no significant differences between DM and salivary concentrations of IL-6, CRP, and CXCL-10 within the non-SARS-CoV-2, mild SARS-CoV-2, and severe SARS-CoV-2 groups (data not shown).

### 3.7. Effect of HT on Concentrations of Salivary IL-6, CRP, and CXCL-10

**Figure 6:**
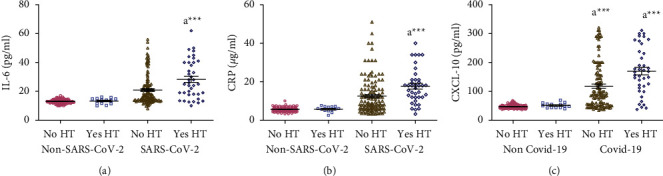
(a-c) Effect of hypertension (HT) on concentrations of salivary IL-6, CRP, and CXCL-10 measured in non-SARS-CoV-2 (*n* = 142) and SARS-CoV-2 (*n* = 158) participants. Data were graphed as a scatter plot vertical with mean ± SE. ^a^Significant difference compared to patients without HT ^*∗∗∗*^*p* < 0.001.

There were no significant differences in the salivary concentrations of IL-6, CRP, and CXCL-10 between participants with and without HT in the non-SARS-CoV-2 participants. On the other hand, salivary concentrations of IL-6, CRP, and CXCL-10 were significantly increased (*p* < 0.001) in SARS-CoV-2 patients with HT compared to SARS-CoV-2 patients without HT (Figures 6(a)–6(c)).

Statistical analysis of data showed no significant differences between HT and salivary concentrations of IL-6, CRP, and CXCL-10 within the non-SARS-CoV-2, mild SARS-CoV-2, and severe SARS-CoV-2 groups (data not shown).

## 4. Discussion

COVID-19 is a fatal respiratory illness caused by the SARS-CoV-2 virus. The severity of the COVID-19 disease is related to the triggering of a cytokine storm. The cytokine storm associated with SARS-CoV-2 virus infection is currently believed to be dominated by IL-6, CXCL-10, and infiltrating macrophages. Serum CRP has been identified as a critical inflammatory measure that fluctuates dramatically in severe SARS-CoV-2 virus infection [19–21]. Inflammation, reflected by cytokine storms and CRP in COVID-19 patients, could worsen the disease [22]. Saliva has been studied and described as a possible source of diagnosis for a wide variety of conditions [23]. The current study suggests saliva as a reliable method to detect the severity of SARS-CoV-2 virus infection due to the convenience of taking samples, the potential for self-collection, the low cost, the elimination of special equipment, and the reduced risk of transmission for healthcare professionals [15].

This study included 158 SARS-CoV-2 infection cases with symptoms ranging from mild (72) to severe (86) and 142 healthy participants. IL-6, CRP, and CXCL-10 were measured in the saliva samples of all participants. Saliva IL-6 and CXCL-10 were significantly increased in both mild and severe SARS-CoV-2 patients compared to the control non-SARS-CoV-2 participants. Saliva CRP was significantly increased only in severe SARS-CoV-2 patients. In severe cases, all of the biomarkers were significantly higher than the mild cases. A significant positive correlation was found between salivary IL-6 and both CRP and CXCL-10 in all SARS-CoV-2 patients.

Our results are similar to recently published results, which proposed that patients with COVID-19 could be distinguished from the noninfected people using a panel of 27 cytokines, including chemokines and growth factors. IL-6, IL-5, GCSF, IL-2, TNF-*α*, GMCSF, and IFN-*γ* were identified as COVID-19-related distinguishable cytokines, but only one (IL-12p70) was detected in the noninfected people. The most predictable cytokines for COVID-19 and control were IL-6 and IL-12p70. Results of serum cytokine profiling in patients with severe symptoms and complications showed a cytokine profile that closely paralleled the saliva cytokine profile at the time of infection [24]. The present study results also agree with those of Santa Cruz et al. [25], who reported a significant increase in serum IL-6 concentration along with the COVID-19 disease stage (I, IIa, IIb, and III). However, they only declared a significant increase in serum CRP concentration in stage III of the disease, the most severe stage. Their results also confirmed a positive correlation between serum IL-6 and serum CRP. Our results also agree with Han et al. [22], who found that SARS-CoV-2-infected patients had higher serum concentrations of IL-6 and CRP than healthy controls.

Furthermore, serum IL-6 concentration in SARS-CoV-2-infected patients was significantly increased in the critical cases than in the moderate and severe cases. Compared to patients treated outside the ICU, SARS-CoV-2-infected patients who were eventually admitted to the ICU had significantly higher serum concentrations of IL-6 and CRP [26]. Like our results, Tripathy et al. [27] observed that asymptomatic and mildly symptomatic SARS-CoV-2-infected patients had significantly higher plasma concentrations of IL-6 and CXCL-10 than controls; furthermore, CXCL-10 was significantly higher in mildly symptomatic patients. In contrast to the result of the present study, there were no changes in plasma IL-6 concentration between asymptomatic and mildly symptomatic patients [27].

This study found less saliva concentrations of CRP and CXCL-10 in SARS-CoV-2-infected females than males. Several investigations around the world have suggested a gender discrepancy in the severity and prognosis of patients with SARS-CoV-2 infection due to the mechanisms of viral infection, the immune response to the virus, the establishment of the inflammatory process, and subsequent systemic consequences, including the thromboembolism. COVID-19 has a gender difference in severity, with women having a better prognosis than men, according to epidemiological statistics [28]. Male mice inoculated with SARS-CoV-MA15, which mimics the clinical symptoms of SARS, are more vulnerable to infection than females. The higher susceptibility of male mice was associated with higher virus titers, neutrophil infiltration, and concentrations of proinflammatory cytokines (IL-1, IL-6, and TNF) in the lungs, suggesting a poorer natural and adaptive immune response in males than in females, most likely due to differences in expression and activity of immune-related genes on the *X* chromosome [29].

There were no significant differences in salivary IL-6, CRP, and CXCL-10 concentrations among overweight, obese, and normal weight SARS-CoV-2-infected patients in the mild and severe cases. Similar to our results, McNeill et al. [30] observed no significant association between obesity and serum IL-6 concentration. On the other hand, the same authors indicated that obese people had higher baseline and peak serum CRP concentrations than nonobese people [30]. Frasca et al. [31] also found a significant positive association between BMI and serum CRP concentration in PCR-positive COVID-19 patients.

The present findings showed significantly higher salivary IL-6, CRP, and CXCL-10 in diabetic and hypertensive SARS-CoV-2-infected patients. Diabetes, cerebrovascular disease, and hypertension are among the most prevalent comorbidities noted in patients with confirmed COVID-19 [32]. Metabolic diseases, including type 2 DM and HT, are associated with chronic low-grade systemic inflammation or meta-inflammation. These diseases constitute deadly systemic scenarios for SARS-CoV-2 infection results [33, 34]. Meta-inflammation develops after activating adipose tissue-resident macrophages, promoting the recruitment of M1-polarized macrophages, which is a more proinflammatory phenotype, and increasing the production of proinflammatory cytokines such as TNF-*α*, IL-6 and chemokines both locally and systemically [35]. The number of white blood cells, acute-phase proteins such as CRP, and plasma concentrations of coagulation components (fibrinogen, D-dimers) also increase during inflammation [36]. In a case series from China, hospitalized SARS-CoV-2-infected cases with DM as the only comorbidity had high CRP and IL-6 [37]. Compared to normoglycemia, diabetes was significantly associated with severe COVID-19, while prediabetes was not a risk factor. CRP mediated 32.7% of the total association between diabetes and severe COVID-19 outcome [38]. The pathogenic link between diabetes and COVID‐19 severity can be described by the following mechanisms: (i) immune dysfunction; (ii) reduced viral clearance; and (iii) heightened inflammatory state [39, 40]. Diabetes is associated with higher CRP levels, [39] similar to our findings. Chronic hyperglycemia is known to incite a proinflammatory, prooxidative state, which is linked to adverse outcome in the critical care setting [41, 42]. Hypertension is more common in patients with severe COVID-19 infections. The clinical presentation of severe COVID-19 presentations has been linked to cytokine storm. Given the essential role of inflammation in the development and pathogenesis of hypertension, it is conceivable that hypertension may represent an immunologically vulnerable condition that predisposes patients to more severe presentations [32]. An increased serum level of proinflammatory cytokines such as IL-1, IL-6, IL-8, IL-17, IL-23, TGF-*β*, and TNF-*α* was observed in hypertensive patients [43].

The present data showed a direct relationship between patients' age and IL-6, CRP, and CXCL-10 concentrations in SARS-CoV-2-infected patients. Aging causes a mild, chronic, systemic proinflammatory reaction known as inflammaging, associated with a progressive decline and dysregulation of immune function. Increased concentrations of systemic proinflammatory cytokines such as IL-1, IL-6, and TNF-*α* characterize inflammaging [44]. Inflammaging creates a favorable setting for developing a cytokine storm in the elderly [34].

Within each COVID-19 disease category, there were no significant differences in salivary levels for IL-6, CRP, and CXCL-10 due to gender, age, DM, and HT differences (data not shown). Accordingly, the effects of gender, age, DM, and HT on the salivary levels of IL-6, CRP, and CXCL-10 observed in SARS-CoV-2 patients might be consequences of their impacts on COVID-19 disease severity.

In conclusion, the present study findings revealed that salivary concentrations of IL-6, CRP, and CXCL-10 measured in SARS-CoV-2 infected patients are comparable to those previously assessed in the blood. The saliva concentrations of these inflammatory biomarkers are associated with SARS-CoV-2 infection severity. Inflammatory markers were also higher in males, patients with diabetes, individuals with high blood pressure, and older patients. In the future, saliva could be used to assess various biomarkers of inflammation related to SARS-CoV-2 infection as a noninvasive diagnostic fluid.

The current study had certain drawbacks. First, the number of patients was small, but a post hoc power analysis utilizing the values obtained to verify the null hypothesis using the mean and standard deviation indicated that the sample was large enough to give 80% power at a 5% significance level. We did not measure the concentrations of IL-6, CRP, or CXCL-10 in the serum, and it is best to do so simultaneously with the saliva sample. A combination of both saliva and serum estimation of those biomarkers will provide more information regarding the correlation between systemic and local events associated with the viral infection and help to prove if this technique could be used in the future as a noninvasive tool to differentiate between different stages of SARS-CoV-2 infection for better selection of management plan. Furthermore, knowing the serum values would have been proper to evaluate which markers are the best in the two biological fluids.

## Figures and Tables

**Table 1:** Demographic and clinical data of the study participants.

Characteristic	Frequency (*n* and %)			*p* value
	Non-SARS-CoV-2 (*n* = 142)	Mild SARS-CoV-2 (*n* = 72)	Severe SARS-CoV-2 (*n* = 86)	Mild against severe SARS-CoV-2
Nationality				
Saudi Arabian	113 (79.6%)	43 (59.7%)	44 (51.2%)	
Non-Saudi Arabian	29 (20.4%)	29 (40.3%)	42 (48.8%)	
Sex				
Male	66 (46.5%)	42 (58.3%)	75 (87.2%)	^*∗∗∗*^ *p* < 0.001
Female	76 (53.5%)	30 (41.7%)	11 (12.8%)	
Age (years)				
<20	2 (1.4%)	3 (4.2%)	0 (0%)	
20–40	72 (50.7%)	50 (69.5%)	12 (14.0%)	
40–60	65 (45.8%)	12 (16.7%)	50 (58%)	^*∗∗∗*^ *p* < 0.001
60–80	3 (2.1%)	6 (8.3%)	24 (28%)	^*∗∗∗*^ *p* < 0.001
>80	0 (0%)	1 (1.3%)	0 (0%)	
BMI (kg/m^2^)				
Under weight (< 18.5)	1 (0.7%)	1 (1.3%)	1 (1.2%)	
Normal weight (18.5–24.9)	43 (30.3%)	25 (34.8%)	10 (11.6%)	
Over weight (25–29.9)	60 (42.3%)	22 (30.7%)	33 (38.4%)	^*∗∗*^ *p* < 0.01
Obese (≥30)	38 (26.7%)	23 (31.9%)	39 (45.3%)	^*∗∗*^ *p* < 0.01
Missing	0 (0%)	1 (1.3%)	3 (3.5%)	
Chronic diseases				
No diseases	108 (76.0%)	48 (66.7%)	22 (25.6%)	
Diabetes mellitus (DM)	15 (10.6%)	6 (8.3%)	44 (51.2%)	^*∗∗∗*^ *p* < 0.001
Hypertension (HT)	14 (9.6%)	7 (9.7%)	32 (37.2%)	^*∗∗∗*^ *p* < 0.001
Bronchial asthma	5 (3.5%)	9 (12.5%)	12 (14%)	
Others	9 (6.3%)	12 (16.7%)	18 (20.9%)	

Data were presented as frequency (*n*) and percentage (%).

## Data Availability

All data used to support the findings of the study can be obtained from the corresponding author upon request.
